# Case Report: Somatic malignancy classified as Wilms tumor arising within an immature teratoma

**DOI:** 10.3389/fonc.2025.1565865

**Published:** 2025-06-17

**Authors:** Emily Muñoz, Cyrus Washington, Pasquale Benedetto, Ali Saad, Oleksandr N. Kryvenko, Dalissa Tejera, Macarena Ines De La Fuente, Michael Ivan, Gregory Azzam

**Affiliations:** ^1^ Jackson Memorial Hospital, University of Miami, Miami, FL, United States; ^2^ Sylvester Comprehensive Cancer Center, University of Miami, Miami, FL, United States; ^3^ Department of Radiation Oncology, University of Miami, Miami, FL, United States; ^4^ Department of Hematology and Oncology, University of Miami, Miami, FL, United States; ^5^ Department of Pathology and Laboratory Medicine, University of Miami, Miami, FL, United States; ^6^ Desai Sethi Urology Institute, University of Miami, Miami, FL, United States; ^7^ Department of Neurology, University of Miami, Miami, FL, United States; ^8^ Department of Neurosurgery, University of Miami, Miami, FL, United States

**Keywords:** Wilms tumor, immature teratoma, non-geminomatous germ cell tumor, somatic malignancy, neuro-oncology

## Abstract

Nongeminomatous germ cell tumors (NGGCTs) are aggressive malignancies known for their rapid metastatic potential. Teratomas, a subtype of NGGCTs, can be classified as either mature (benign) or immature (malignant). Immature teratomas carry a higher metastatic risk than mature teratomas due to their embryonic-like tissue composition. Intracranial teratomas are rare in nature and can develop secondary malignancies, such as Wilms tumors. We report the case of a 70-year-old man with a history of prostate cancer who presented with neurological symptoms and was diagnosed with a Wilms tumor arising from an immature teratoma. A heterogenous morphology, including squamous, cartilaginous, and neural differentiation, was revealed upon surgical resection. Despite interventions, the patient experienced rapid disease progression and eventually passed away in hospice care 7 months after the initial diagnosis. This case highlights the complexity of diagnosing and managing NGGCTs, particularly when secondary malignancies arise. Ultimately, it underscores the need for careful diagnosis and precise therapeutic strategies to manage these tumors.

## Introduction

Nongeminomatous germ cell tumors (NGGCTs) are a type of testicular cancer that develops from the germ cells responsible for sperm production. These tumors are generally more aggressive than pure geminomas, another type of germ cell tumor, and tend to spread more rapidly. NGGCTs consist of several subtypes: embryonal carcinoma, which is highly malignant and fast-growing; yolk sac tumors, more commonly found in children; choriocarcinoma, a rare but highly aggressive type; and teratomas, which can be either mature or immature, and frequently occur in the ovaries of women and the testicles of men (1). Mature teratomas are typically benign and composed of well-differentiated tissues, whereas immature teratomas are malignant, containing less differentiated, embryonic-like tissues that pose a higher risk of metastasis. Immature teratomas consist of three germ cell layers: ectoderm, endoderm, and mesoderm (2).

The pluripotency of teratomas arises from their undifferentiated cells, which have not yet specialized into specific cell types. As these cells are in an early developmental stage, they can differentiate into various tissue types, including those found in different organs and structures of the body, such as bone, muscle, hair, and even neural tissue. This undifferentiated state enables teratomas to form diverse and complex tissues, highlighting their pluripotent nature (3).

Intracranial teratomas, or teratomas in the brain, are relatively rare, accounting for less than 1% of all intracranial tumors (4). These tumors can be congenital, meaning they are present at birth, and are often found in the pineal or suprasellar region of the brain. Malignant intracranial teratomas may include an additional malignant component of a conventional somatic type, characterized by a population of atypical cells. This component often exhibits a nodular or infiltrative pattern and can consist of carcinoma, sarcoma, primitive neuroectodermal tumors, nephroblastoma, melanoma, or neuroendocrine tumors (5, 6).

An immature teratoma is the only type of germ cell tumor graded based on immature neural features, which helps predict overall survival (7). This grading system is known as the Norris grading system (8). Histopathologically, immature teratomas are characterized by clusters of poorly differentiated, primitive, and blast-like cells. The most distinctive features of these tumors are the immature neuroectodermal elements, which include groups of neuroblasts, highly active immature glial cells, and primitive pigmented retinal tissue (9).

The degree of immaturity and the type of tissues involved are essential in determining the grade of the tumor, which is directly related to its malignancy potential. Immature teratomas are considered malignant primarily due to their potential for aggressive growth and metastasis. Microscopically, the three embryonic layers in immature teratomas often include neuroepithelium, which contributes to the tumor’s malignancy and serves as a negative prognostic indicator (10). The grading of the tumor, based on the amount and type of immature tissue, helps predict its aggressive nature. Higher-grade tumors contain more immature tissue and are more likely to exhibit malignant behavior, increasing the risk of metastasis. Secondary malignancies in immature teratomas can include neuroectodermal-type tumors, yolk sac tumors, and primitive neuroectodermal tumors. These cancers may be nongerm cell types and represent a category of secondary cancers that can develop within teratomas (11). Malignant germ cell tumors (GCTs) may transform into nongerm cell cancer types resembling those found in other organs, known as somatic-type malignancies (SMs) (12).

Nephroblastoma, also known as Wilms tumor (WT), is a malignant kidney tumor typically found in children. However, it is rare in adults, accounting for only 0.5% of all kidney tumors, with an incidence rate of 0.2 cases per million (13). Adults represent only 3% of reported nephroblastoma cases. Brain metastasis is uncommon, occurring in 1%–2% of cases in children and is virtually unknown in adults due to its rarity (14). Primary Wilms tumor of the brain is exceedingly rare.

WT typically comprises three histological components: the blastema, consisting of primitive, undifferentiated cells resembling fetal kidney tissue; the stroma, which contains connective tissue that may be fibrous or myxoid; and the epithelium, resembling immature kidney tubules and glomeruli. The blastema is typically the predominant and most critical component for diagnosis, while the stroma and epithelium provide additional context for the tumor’s differentiation and overall characteristics (15).

The purpose of this study is to present a rare case of Wilms tumor developing within an immature teratoma in an elderly patient.

## Case report

The patient is a 70-year-old African American man with a medical history of diabetes mellitus, hypertension, congestive heart failure, coronary artery disease, and substance use disorder. One year prior to this case presentation, the patient was diagnosed with stage IIB prostate cancer after elevated prostate-specific antigen (PSA) levels were detected. One month following the diagnosis, he was considered a poor surgical candidate and began treatment with intensity-modulated radiation therapy (IMRT) to a total tumor dose of approximately 7,000 cGy in 28 fractions, along with androgen deprivation therapy (ADT). He completed radiation therapy for prostate cancer in October 2021. ADT consisted of a 6-month course of Degarelix, followed by Lupron.

**Figure 1:**
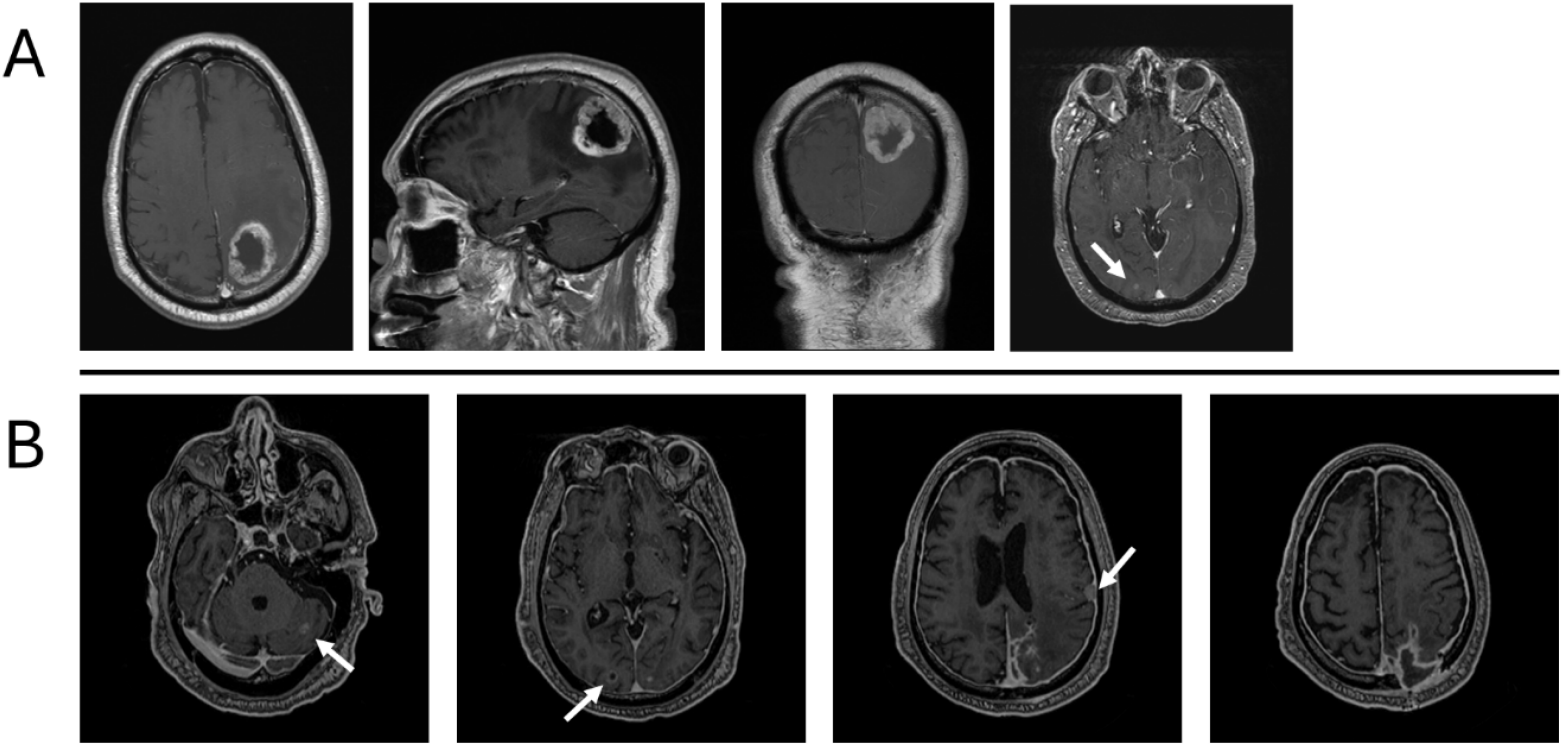
Description of the primary tumor before removal at presentation in August 2022 **(A)** and recurrence in February 2023, without adjuvant chemotherapy or radiation therapy **(B)**. **(A)** Large, centrally necrotic, peripherally enhancing mass centered in the left parietal lobe with marked surrounding edema. Additional punctate foci of enhancement in the right parieto-occipital lobe. **(B)** Peripheral enhancing lesion in the right occipital lobe measuring 8 mm; left cerebellar enhancing lesion measuring 1 cm; extra-axial enhancing lesion along the left temporal convexity measuring 1.2 cm; diffuse pachymeningeal enhancement.

In August 2022, the patient presented to the emergency department with generalized weakness, abnormal gait, and delirium. An MRI of the brain with and without contrast demonstrated a left occipitoparietal mass with a large area of surrounding vasogenic edema (**Figure 1**). To rule out metastatic disease, the patient underwent a computed tomography (CT) scan of the chest, abdomen, and pelvis, which revealed a cavitary lesion in the left upper lobe, more suspicious for infection than malignancy.

**Figure 2:**
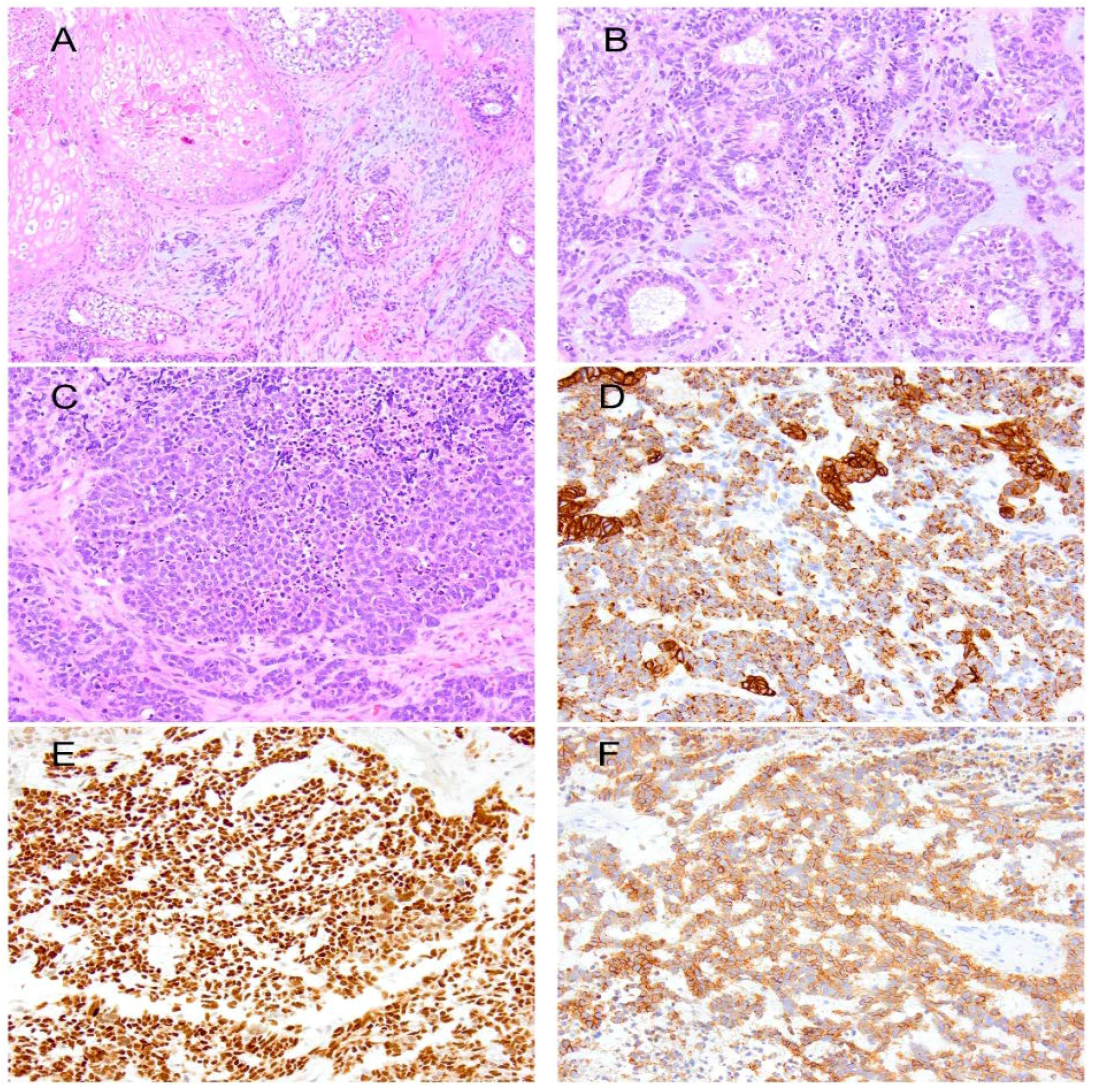
**(A)** Immature teratoma showing squamous epithelium, cellular spindle cell stroma, and scattered foci of immature neural components. **(B)** Wilms tumor forming tubules in a background of blastema. **(C)** Solid epithelium in the Wilms tumor. Immunohistochemically, the primitive and tubular epithelium are positive for keratin 8/18 **(D)**, PAX8 **(E)**, and CD56 **(F)**.

The patient was started on dexamethasone 4 mg for edema and Keppra 1 mg twice a day for seizures. Before resection, he underwent a CT scan of the chest, abdomen, and pelvis to assess for evidence of primary malignancy, and these studies were negative. Preoperative MRI demonstrated a peripherally enhancing, centrally necrotic mass in the left parietal lobe measuring 4.1 cm × 3.7 cm × 4.4 cm (AP × TV × CC) with significant peritumoral edema and mass effect with shift. An enhancing lesion in the right occipital lobe measuring 5 mm was also observed and deemed stable. He underwent a left parietal craniotomy for resection of the tumor using a neuronavigation microscope. Multiple specimens were sent for pathological assessment, revealing that the tumor exhibited variable morphology, including squamous, cartilaginous, neural, and skeletal muscle differentiation. An overgrowth of a more immature component, characterized by solid malignant cell growth, tubular formation, and regions reminiscent of blastemal, was observed. Brisk mitotic and apoptotic activity was noted in this immature component. Immunohistochemically, the immature component was positive for keratin 8/18, pan-cytokeratin AE1/AE3, CD56, thyroid transcription factor 1 (TTF1), and paired-box gene 8 (PAX8). A small subset of cells was positive for insulinoma-associated protein 1 (INSM1), synaptophysin, myogenin, myogenic differentiation 1 (MyoD1), tyrosine-protein kinase KIT, CD117, and spalt-like transcription factor 4 (SALL4). The tumor was best classified as a Wilms tumor arising within an immature teratoma (**Figure 2**). The operative report noted an unusual dural lining on the inner surface of the dura, extending well beyond the actual tumor. This lining was removed and debulked, then carefully excised medially and posteriorly, although gross total resection was achieved.

Postoperative MRI of the brain with and without contrast revealed expected postoperative changes. In addition to pneumocephalus in the anterior cranial fossa, there was interval development of bilateral frontal subdural collections and hematomas, with increased FLAIR signal abnormality measuring up to 4 mm in the right frontal lobe and 3 mm in the left frontal lobe was observed. No change was observed in the degree of vasogenic edema in the left parietal lobe, which continued to exhibit mild mass effect and a left-to-right midline shift. A 5-mm focus of enhancement was observed in the right occipital lobe. The patient was admitted for 2 weeks postoperatively, during which he completed inpatient physical therapy.

His case was discussed at the neuro-oncology interdisciplinary tumor board, where a consensus was reached to recommend craniospinal irradiation (CSI). The tentative radiotherapy prescription included 3,600 cGy in 20 fractions, followed by a boost to 5,400 cGy to the tumor cavity in 10 additional fractions using intensity-modulated proton therapy (IMPT). An exploratory objective of the ongoing ANCS2021 trial—”A Phase II Study of Chemotherapy Followed by Radiation Therapy for Children and Young Adults with Localized Non-Germinomatous Germ Cell Tumors of the Brain or Spinal Cord”—is to prospectively compare outcomes based on radiation modality, photon versus proton. Domains include cognitive, social, behavioral, auditory, and neuro-endocrine functioning after radiation. Previous studies have shown that using protons for spinal or paraspinal sites can significantly decrease the dose to the thyroid, heart, lungs, esophagus, spinal cord, kidneys, bowel, bone marrow, and/or reproductive organs (16). For patients receiving CSI, proton therapy versus photon therapy is associated with less weight loss during treatment, a lower incidence of grade 2 nausea and vomiting (26% vs. 71%), and less esophagitis during therapy (57% vs. 5%) (17). Smaller WBC, platelet, and RBC counts have also been observed in patients undergoing CSI with protons compared to photons (17). Therefore, proton therapy is offered at our facility for patients with CNS malignancies requiring CSI as part of definitive treatment. The patient was discharged with a plan to receive definitive radiation therapy with protons and a 1-month follow-up with neurosurgery in the outpatient setting. At the initial radiation oncology consultation, laboratory tests for alpha-fetoprotein (AFP) and the human chorionic gonadotropin (hCG), as well as PET imaging and spinal MRI, were ordered to aid in the initial workup and treatment planning. Before these studies could be performed and radiation therapy initiated as planned, the patient was lost to follow-up.

In November of 2022, 3 months after the initial diagnosis, the patient presented to the emergency department with complaints of worsening weakness and lethargy, which had begun 3 days prior. Upon admission, a CT of the brain without contrast and an MRI of the brain with and without contrast were performed. CT imaging demonstrated postsurgical changes from a left craniotomy for resection of a left parietal mass. A thin curvilinear hyperdensity was noted at the anterior aspect of the suspected mixed-density mass, measuring 5.1 cm at the surgical bed. Findings were concerning for tumor recurrence. MRI revealed a new, large, heterogenous enhancing lesion along the left parietal lobe/surgical bed, suspicious for recurrent tumor. The mass measured about 4.9 cm × 3.5 cm × 3.8 cm. Also noted were a peripherally enhancing lesion in the right occipital lobe and a lesion in the cerebellum (**Figure 1**). At this point, the patient underwent restaging with CT of the chest, abdomen, and pelvis, which were negative for metastatic disease. He subsequently underwent resection of the recurrent tumor but developed postoperative complications, including infection. He declined radiotherapy as offered in August 2022, as he was planning to relocate out of state.

In February of 2023, 2 months after relocating, he presented to an external facility with an acute onset of right facial droop, garbled speech, and right-sided weakness. Brain MRI with and without contrast revealed numerous mass lesions concerning for dural metastasis along the extra-axial spaces adjacent to the lateral convexity of the left frontal lobe and the posterior aspect of the left parietal lobe. The lesion along the lateral convexity of the left frontal lobe measured approximately 3.8 cm × 2.3 cm × 3.5 cm. There was a conglomeration of extra-axial lesions along the left high parietal lobe, extending through the adjacent calvarium into the subcutaneous soft tissue of the scalp. In aggregate, these lesions measured approximately 3.8 cm × 5.1 cm × 8.6 cm. Heterogeneous contrast enhancement was observed in the adjacent left parietal lobe, extending into the subcortical white matter, raising concern for intraparenchymal metastasis extension. Additionally, diffuse dural thickening and contrast enhancement were present, concerning for widespread metastatic disease involvement. Additional peripherally contrast-enhancing intraparenchymal lesions were observed in the right posterior occipital lobe, measuring 1.2 cm × 1.6 cm × 1.5 cm. Another metastatic lesion was identified in the left cerebellum, measuring approximately 1.5 cm × 1.7 cm × 1.5 cm. Beyond the extension of metastatic disease to the left parietal calvarium, an additional osseous metastasis was present in the right eccentric frontal bone, measuring approximately 1.2 cm × 1.4 cm, with apparent extension into the adjacent scalp. Due to the surrounding vasogenic edema, predominantly within the left parietal lobe, and the mass effect from the extra-axial lesions, there was effacement of the left lateral ventricle and approximately 4 mm of rightward midline shift.

Radiation oncology discussed his case at the interdisciplinary neuro-oncology tumor board conference, where whole brain radiation therapy was considered due to the patient’s rapid functional decline. However, given his neurological status with encephalopathy, oncology determined that he would not be able to tolerate the treatment. After discussing options with his family, the patient was ultimately enrolled in hospice care. He passed away shortly thereafter, 7 months after diagnosis.

## Discussion

A rare occurrence in oncology is the development of a Wilms tumor from an immature teratoma. Somatic malignancies arising from germ cell tumors are relatively uncommon, occurring in approximately 2.7% to 8.6% of germ cell tumor cases, and are more frequently observed in late relapses (18–20). Central nervous system GCTs are classified into three main categories: germinomatous, nongerminomatous, and mixed GCTs (21). These tumors are relatively rare, comprising only 0.3% to 0.6% of all intracranial tumors (22).

The standard protocol for the diagnostic workup of central nervous system germ cell tumors includes MRI of the brain and spine, measurement of tumor markers (β-hCG and AFP) in both serum and cerebrospinal fluid (CSF), and histological confirmation via biopsy (23–25). Treatment planning and prognosis heavily depend on the lesion’s histopathological features and the extent of disease spread. Even in advanced stages, definitive therapy involving a combination of chemotherapy and radiation is administered according to globally accepted guidelines, leading to relatively high recovery rates. ANCS0122, a trial investigating neoadjuvant chemotherapy followed by radiation, reported that the overall 5-year survival rate for all patients with NGGCT exceeded 90% (26). In the present case, it could not be definitively determined whether the patient’s diagnosis represented a primary or metastatic tumor. The primary tumor was identified as an intraparenchymal mass in the left parietal lobe, which is not an uncommon location for NGGCTs. However, imaging studies suggest no other primary malignancy site, although nuclear imaging was not performed. In the event that the tumor was primary, the neuro-oncology interdisciplinary tumor board recommended proceeding with CSI. The tentative radiotherapy prescription included 3,600 cGy in 20 fractions, followed by a boost of 5,400 cGy to the tumor cavity in 10 additional fractions using IMPT. Unfortunately, this patient was lost to follow-up and did not receive adjuvant radiation. Given the multifocal recurrence and meningeal involvement, the suggested CSI regimen, in combination with chemotherapy, appears appropriate for similar cases.

The prognosis for these tumors depends heavily on complete surgical resection and a combination of radiotherapy and chemotherapy. Prior to the introduction of cisplatin-based chemotherapy in addition to radiation therapy, CSI alone yielded 5-year survival rates of 30%–50%, with most recurrences occurring within 18 months (26). A European study, the SIOP-CNS-GCT-96 trial, utilized high-dose chemotherapy followed by risk-adapted radiotherapy in patients with intracranial NGGCTs. In this trial, nonmetastatic patients received four courses of cisplatin/etoposide/ifosfamide followed by focal radiotherapy (54 Gy). In a cohort of 116 patients with localized malignant NGGCT, the 5-year progression-free survival and overall survival rates were 72% and 82%, respectively. Primary tumor sites included 67 pineal, 35 suprasellar, five bifocal, and nine at other locations. Twenty-seven patients experienced relapse, and 17 of them died from the disease. In conclusion, the SIOP-CNS-GCT-96 trial demonstrated that patients with localized intracranial NGGCT can achieve adequate disease control using focal radiotherapy fields (5-year PFS of 72%) when combined with high-dose chemotherapy (27). In the same study, patients with metastatic disease involving the craniospinal axis received chemotherapy followed by CSI to a total dose of 30 Gy, with an additional 24 Gy boost to the primary tumor and macroscopic metastatic foci. These patients had a 5-year overall survival rate of 75% (27).

Few cases of immature teratomas developing secondary malignancies have been reported in the literature. Cheema et al., in a case study on teratomas with somatic-type malignancies, described a 44-year-old man who relapsed with an NGGCT that had transformed into adenocarcinoma. Initially treated for testicular cancer comprising teratoma, embryonal carcinoma, and yolk sac components, the patient later developed adenocarcinoma in an enlarged retroperitoneal lymph node, confirmed as a somatic-type malignancy arising from the original germ cell tumor (28). The primary treatment detailed was surgical resection of the tumor, followed by regular monitoring. He discontinued chemotherapy consisting of etoposide and cisplatin after one cycle due to poor tolerance.

In another case study, Georgia reports a rare case of an intracranial immature teratoma with secondary malignant transformation in a 35-year-old man. The patient initially presented with seizures and a left fronto-basal tumor, which was surgically excised. Histopathological analysis revealed a complex tumor with both mature and immature elements, including a significant presence of primitive neuroectodermal tubules. After surgery, the patient experienced rapid recurrence on the contralateral side, characterized by the overgrowth of a primitive neuroectodermal tumor (PNET)-like component, indicative of malignant transformation. The patient underwent chemotherapy and radiotherapy, leading to a favorable outcome with no relapse over 6 years (22).

Additional case studies have supported the adjunctive role of radiation therapy in managing teratomas following malignant transformation or in cases of residual disease. In one such case, a 36-year-old woman with an incompletely resected mediastinal carcinoid teratoma underwent adjuvant radiotherapy with a total dose of 50 Gy. The treatment effectively stabilized the residual tumor, with no progression reported after 2 years of follow-up. This suggests that postoperative radiotherapy can contribute to long-term disease control when complete resection is not achievable (26).

Similarly, a case involving a 73-year-old woman with stage IV malignant transformation of a mature cystic teratoma into squamous cell carcinoma demonstrated the utility of concurrent chemoradiotherapy as a nonsurgical option. Due to her comorbidities and poor tolerance for surgery, she was treated with a combination of chemotherapy and radiation. This approach led to disease suppression and improved quality of life, underscoring the potential of CCRT in managing aggressive, transformed teratomas in medically inoperable patients (27).

Although radiation therapy is not standard for mature teratomas, it may be considered in specific scenarios, such as when malignant transformation occurs or in cases of recurrence where complete surgical resection cannot be performed. Studies have reported its use as an adjunct therapy in intracranial teratomas with aggressive histologic components or incomplete resection, particularly when somatic-type malignancies, such as sarcoma, PNET, or Wilms tumor, are involved. In these cases, the administration of radiation therapy with techniques like IMRT or proton therapy has proven valuable for local control and may potentially improve outcomes. However, given the rarity of these tumors and the lack of prospective trials, management decisions are often based on multidisciplinary consensus and extrapolated from similar entities. Therefore, further studies and long-term follow-up data are needed to better define the role of radiotherapy in managing both mature and immature teratomas with malignant transformation.

## Conclusion

Few cases of Wilms tumor arising within an immature teratoma have been reported in the literature. Gross total resection followed by adjuvant radiation therapy is recommended after diagnosis, provided the patient’s clinical condition allows. This case underscores the complexity of managing NGGCTs, particularly when secondary malignancies such as Wilms tumor develop within teratomas. It highlights the critical need for careful diagnosis and precise treatment strategies in addressing these rare and aggressive tumors.

## Data Availability

The original contributions presented in the study are included in the article/supplementary material. Further inquiries can be directed to the corresponding author.
